# Supplementary figures and images for: ECG performance in simultaneous recordings of five wearable devices using a new morphological noise-to-signal index and Smith-Waterman-based RR interval comparisons
Source: PLoS One. 2022 Oct 5;17(10):e0274994. doi: 10.1371/journal.pone.0274994 (PMC9534432; doi:10.1371/journal.pone.0274994)

nstdb/118e00 with 0 dB Signal-to-Noise Ratio

Noisy parts

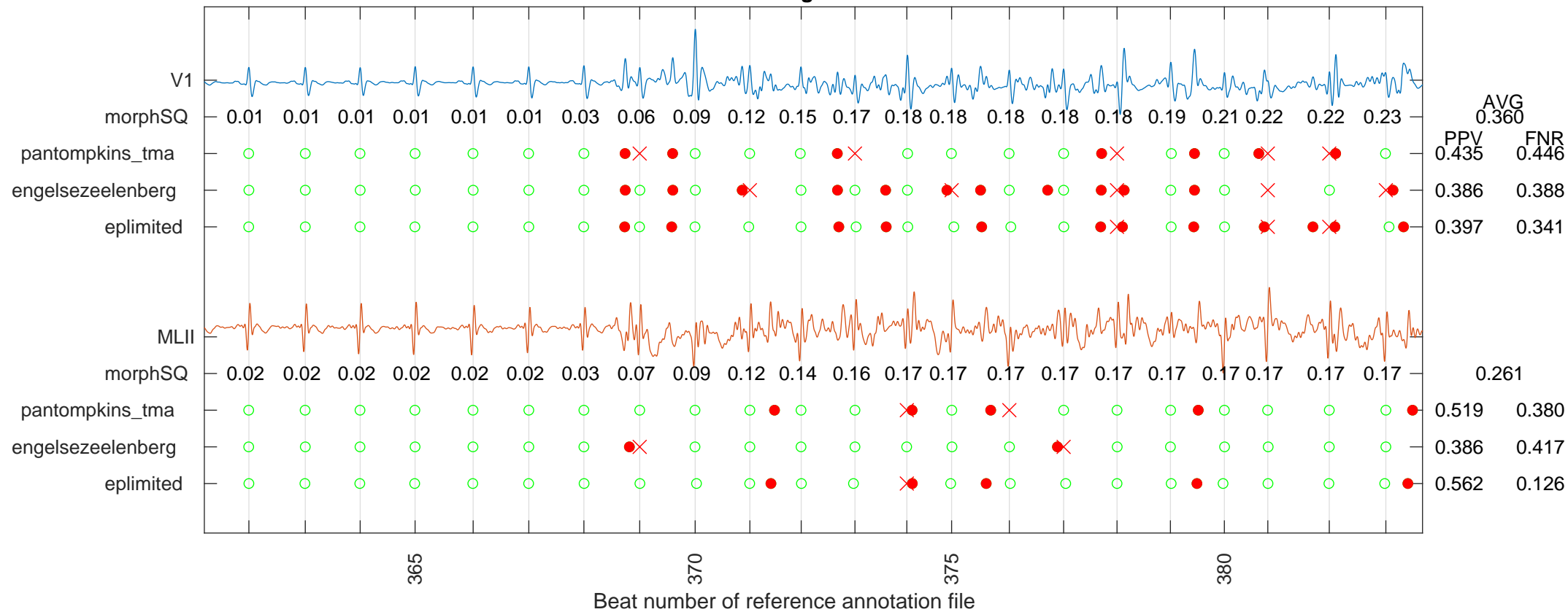

Supplement: S2 Fig — (PDF) [file pone.0274994.s002.pdf]

annotator eplimited engelsezeelenberg pantompkins\_tma

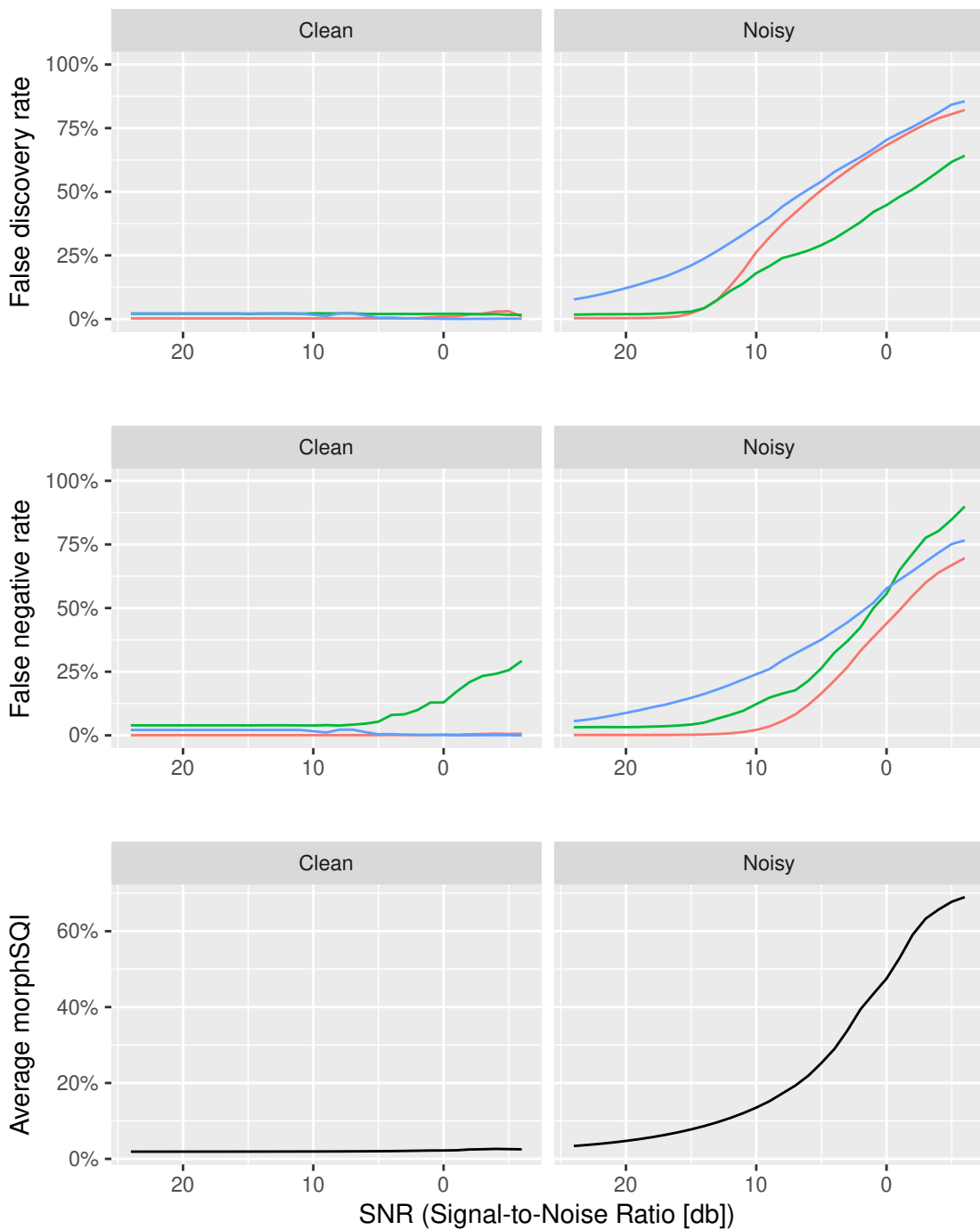

Supplement: S3 Fig — (PDF) [file pone.0274994.s003.pdf]

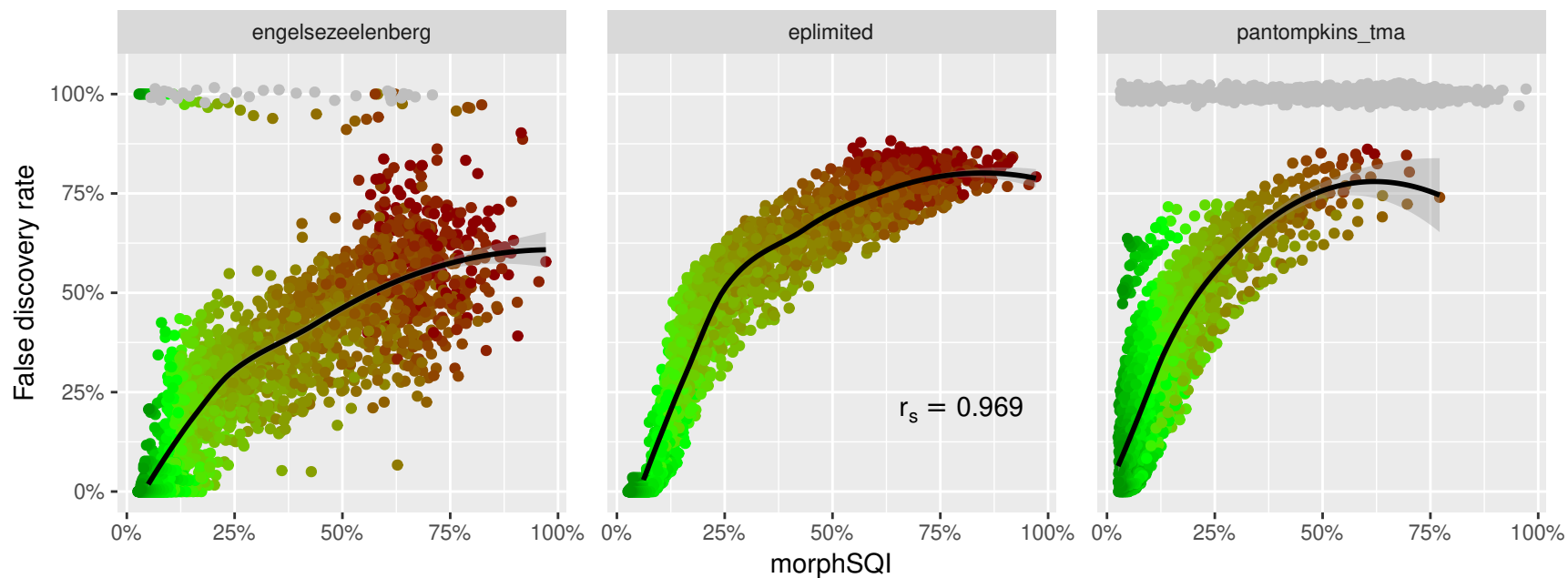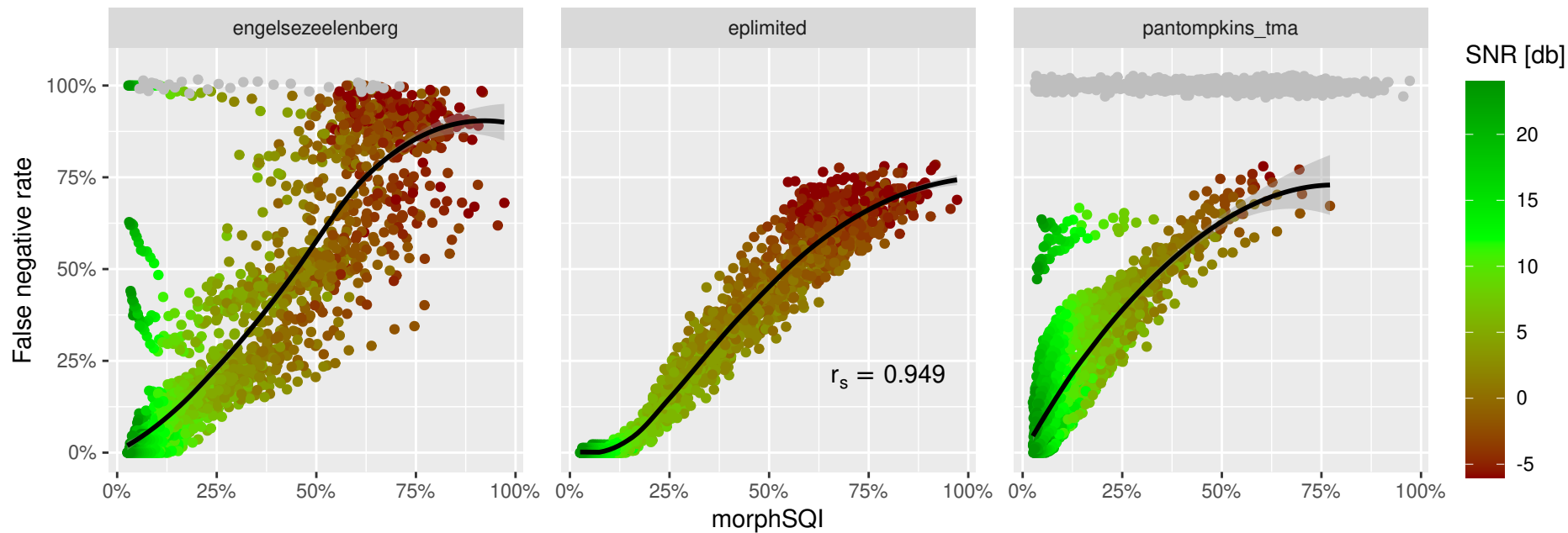

Supplement: S4 Fig — (PDF) [file pone.0274994.s004.pdf]

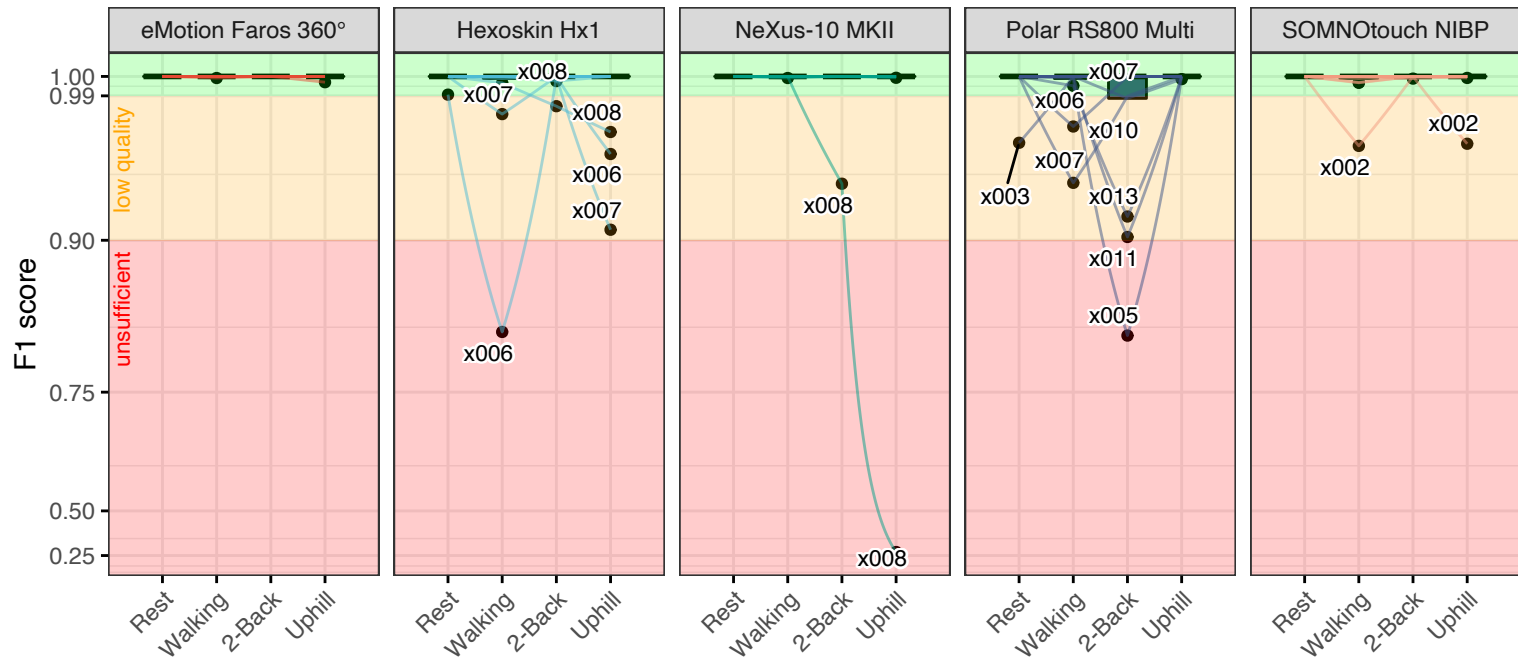

Supplement: S5 Fig — (PDF) [file pone.0274994.s005.pdf]
