# Supplementary material for: ECG performance in simultaneous recordings of five wearable devices using a new morphological noise-to-signal index and Smith-Waterman-based RR interval comparisons
Source: PLoS One. 2022 Oct 5;17(10):e0274994. doi: 10.1371/journal.pone.0274994 (PMC9534432; doi:10.1371/journal.pone.0274994)
Supplement: Attachment — Submitted filename: Response_to_Reviewers.pdf [file pone.0274994.s006.pdf]

Response to Reviewers

Dear Reviewers,

Thank you very much for your thoughtful comments. They allowed us to improve the overall quality of the manuscript and we hope that our now improved version will fit your entitlements.

Response to Reviewer 1:

Reviewer #1:

Dear Reviewer 1, thank you very much for taking the time to comment on our manuscript. We thoughtfully tried to integrate your points in our new revised version of our manuscript. Please find our comments and adjustments below.

However, I have concerns regarding
i) the fact that this work introduces a novel signal quality measures without evaluating it or comparing it to other algorithms;
ii) the cited references as important up-to-date literature is not considered;
iii) the document content and structure being sometimes chaotic and not following IMRAD.
Moreover, iv) at multiple points in the work the wording is not uniform, the title requires changes and v) the level of detail is too superficial at some points.

Thank you for mentioning those valid points. We tried to address all of them and you'll find a detailed explanation of our improvements in the following sections.

i) The authors propose a new heuristic algorithm for ECG signal quality estimation. I am completely fine with that but this requires to evaluate its performance w.r.t. state-of-the-art methods in this field and/or test/synthetic signals with a pre-defined signal quality. Unfortunately, both are missing which prevents to assess the quality of the proposed method in any way.

I suggest the authors to either i) apply a state-of-the-art method to their data and compare the results to the results achieved with the proposed method or ii) apply their method to synthetic signals with manually added noise (e.g. baseline wander, muscle twitches) at pre-defined signal-to-noise levels (as done in [23]). Ideally, both experiments are conducted.

Thank you for mentioning this problem. To address this problem we added new supporting information and conducted additional analysis using a synthetic signal with artificially added noise (see Figures S3 and S4 for further information). Furthermore, a comparison of different state-of-the-art beat-detection-algorithms was added to address reviewer 2's suggestions. Both approaches were combined in those supporting information/figures.

Figure S3 and S4 summarize the results from the application of the morphSQ-methodology on synthetic signals of the PhysioNet Noise Stress Test (NST) database (https://doi.org/10.13026/C2HS3T). This database contains two original records (total length 30 minutes each) with realistic noise added to clean signals at specific time periods (alternating every two minutes). The ground truth beat annotation files from the clean recordings was used to evaluate the PPV and FNR. We have decreased the step-length to 1 db of the pre-defined signal-to-noise ratio bandwidth (originally 24 to -6 db with a 6 db step-length) to increase resolution, and

improve interpretability through the generation of additional noisy records by applying the nst-function (from the WFDB toolbox, https://doi.org/10.13026/egpf-2788) to 100 relatively clean records of the set-p training database of the PhysioNet/CinC Challenge 2014 (see methodology in https://doi.org/10.22489/CinC.2017.182-178, Challenge data and description: https://doi.org/10.1088/0967-3334/36/8/1629, https://lcp.mit.edu/pdf/MoodyCinC2014.pdf , https://physionet.org/content/challenge-2014/1.0.0/).

Based on the suggestion of Reviewer 2 additional beat-annotation-algorithms were included in the analysis. In a first step 13 different beat-annotation-algorithms were compared for their overall FNR during all four experimental phases of our dataset. In addition to widely used algorithms (wqrs, eplimited, pantomkins, hamilton, engelsezeelenberg) newer approaches like the envelope-based rdeco (https://doi.org/10.13026/x6j7-sp58, https://doi.org/10.7717/peerj-cs.226), or neural-network-based (LSTM) ecg2rr (https://doi.org/10.1145/3341105.3373945) were used. Figure A1 and A2 illustrate the results of these comparisons and are only added for this letter. Best performing algorithms were eplimited, engelsezeelenberg, and pantomkins_tma, those are included in the noise-related analysis (as depicted in Fig. S3 and S4).

Description of Fig S3:
Figure S3 is composed of two parts. Part S3a shows the beat detection performance of the three best-performing algorithms expressed by false discovery rates (FDR) and false negative rates (FNR). Part S3b illustrates the corresponding performance of morphSQI based on reference annotations. Analysis were based on 100 recordings (extended from set-p database) with increasing amount of noise for clean parts and noisy parts separately (left and right column of the figure). The average morphSQI was computed from the same recordings and the relation to the SNR can be observed in the accuracy measures of the three algorithms and in morphSQI at the same time. Correlations of morphSQI with FDR and FNR were further investigated results can be seen in Fig S4.

Description of Fig S4:
Figure S4 show the correlation of the new signal quality metric morphSQ with the beat detection performance of the three detection algorithms evaluated from the noise stress test recordings.
We have noticed that for some of the recordings the algorithm from Engelse-Zeelenberg and the modified Pan-Tompkins were not able to detect any beat. This resulted in an empty annotation file and hence FDR and FNR cannot be computed. As all beats are missed, we set FDR and FNR to 100% and marked them as gray dots in the scatter plots. Spearman's rank correlation coefficient r_S was computed between morphSQI and FDR/FNR values of eplimited. With a FDR-correlation of r_S = .969 and a correlation to FNR of r_S = .949 a strong relation of beat detectability and morphSQI can be assumed. Compared to FDR and FNR morphSQ has the advantage to be possibly computed locally (from a few beats only) whereas the estimation of FDR and FNR usually rely on at least dozens of beats. Following these observations and analysis we assumed an increased reliability and robustness of eplimited and thus used it for further analysis of our experimental data (see Tab. 4).

Further a short paragraph was added to the manuscript covering this topic (see page 9 line 287):

For a better understanding of morphSQ, Fig 2 shows different ECG samples and the corresponding morphSQ (additional cases are illustrated in Fig S2). Using clean ECG recordings and realistic noise measurements from PhysioNet we investigated the performance of beat detection and morphSQ in relation to signal noise. Fig S3 shows an example ECG from the Noise Stress Test database, where realistic noise was added to a clean record. morphSQ has been

computed at each beat and the performance of beat detection in clean and noisy ECG sections was evaluated. The relation of average morphSQ with increasing SNR ratios was investigated using the methodology as described in Vollmer 2017. Fig S4 summarizes the results and shows the direct relation to NSR. The similarity-based signal quality expressed by morphSQ is sensitive to beat misplacements and therefore requires accurate, complete, and verified beat annotations.

This would lead the reader to appreciate the many open parameters (e.g. k=8, w=[-2,2], l_qrs=100ms, morphSQ < 10%) because at the moment the reader cannot assess how robust the proposed algorithm and results are.

We like to address this point in an additional section. Even though we would like to systematically manipulate all the parameters to give the reader a better understanding of morphSQ the basic intention during the development was to rather preset those parameters than to test them systematically. The chosen values for k, ω, l_{qrs} and morphSQ were based on theoretical assumptions and mathematical necessities. We like to offer a broader description of those assumptions and necessities in the following section of this response letter and added a short paragraph covering this topic in our manuscript in line 274 on page 9.

A) The parameter k indicates the number of beat cycles from which the stationary median beat cycle will be computed. For the choice of k two aspects have to be considered: k has to be as low as possible so morphSQ can still be seen as a local index (and to allow morphological changes of the physiological signal during long-term measurements), while at the same time being as great as necessary to assess a reliable value of dispersion. As a central dispersion parameter, k has to be element of all even natural numbers to take the same number of cycles left or right into account. k=8 was chosen to meet both conditions to be as low as possible while offering the chance of a valid comparison. With k=8 (looking at 4 cycles left and right, respectively) and a heart rate of 60 bpm, a time frame of approximately 9 seconds is considered which can still be seen as a local indicator, especially compared to FNR or FDR. Increasing k leads to a two second extension of the time frame for each step k is increased.
B) Weighting by the notched Gaussian distribution (parameter ω) improves the nature of locality, as each frame that is further away from the central beat location (outer beat cycles) would get a lower weight than those frames closer to the reference location, which shows the direct relationship between k and ω. ω are values of the Gaussian distribution with μ=0 and σ=2. Using σ<2 would decrease the influence of the outer beat cycles which would have the almost the same effect than reducing k. Using σ>2 would negatively effect the locality as the weights would converge to a uniform distribution (finally a constant) (see Fig. A3 for further information on the influence of ω on the consideration of each beat cycle). The Figure shows the decrease of weights depending on the distance to the central beat location (central R peak located at 0). Each subplot is segmented to highlight 4 RR cycle each left and right. Dashed lines represents the average weight of the corresponding RR cycle.
C) Setting the notching length l_{qrs} to 100ms, was based on physiological assumptions. Main objective was to prevent the quantification of respiration-related modulations of the R peak amplitude, as this is not related to noise. The full QRS sequence usually lasts around 110-120ms in a healthy population. Taking into account, that there might be some minor inaccuracies in R peak detections, a notching length of 100ms seem to be sufficient. A shortage of the time frame would possibly lead to an overestimation of morpSQ due to respiration-related modulations.

D) While k, ω, and l_{qrs} are adjustable parameters to determine morphSQ, the cut-off-value of 10% is used for a binary classification of clean|noisy ECG segments. We have seen that morphSQ values larger than 10% are related to decreased performance rates of standard detections algorithms that might disturb the analysis of experimental data (noise-induced bias)(see Fig. S3). morphSQ values below 10% are usually achieved at low noise levels (SNR>17dB) resulting in excellent performances rather independent of the used heart beat detection algorithm. We explicitly included the rates of morphSQ<10% in Table 3 as it constitutes an easy-to-understand value of signal quality, additionally mean and SD-values are presented as advanced indicators for direct comparison and potential statistical analysis.

If you deem it necessary we can provide these information in combination with Fig. A3 as an additional supplementary material. Here you can find the mentioned additional paragraph:

The used specifications for the parameters k, ω, l_{qrs}, as well as the used cut-off-value of 10% for morphSQ were chosen based on theoretical assumptions and mathematical necessities. Setting k = 8 and weights ω taken from notched Gaussian distribution function were necessary to reinforce the locality of morphSQ. Increasing k or making ω constant would lead to an reduced influence of the momentarily centered beat (R peak). Considering the physiology basis of the QRS complex a notching length of 100 ms was chosen for l_{qrs} to prevent the effect of respiration-related modulations of the R-Peak on morphSQ calculation. The choice of morphSQ values <10% to binary classify the overall signal quality is based on the descending performance of standard heart beat detection algorithms around this cut-off-value (see Fig. S3).

Moreover, the reader cannot get "a feeling" for the proposed morphSQ values. Fig. 3 shows four ideal ECG signals and their corresponding morphSQ values but what about the special cases? What influence does baseline wander, motion artifacts etc. have on the accuracy of morphSQ?

Thank you for mentioning this point. In addition to Fig. 3 we added a new supplementary material Figure S2. This figure illustrates the influence of realistic noise on morphSQ (using the NST function which includes "intervals that contained predominantly baseline wander (in record 'bw'), muscle (EMG) artifact (in record 'ma'), and electrode motion artifact (in record 'em'). The ECG recordings were created by the script nstdbgen-" see PhysioNet NSTDB: https://doi.org/10.13026/C2HS3T). Additionally Figure S2 includes the detected beats of the three best performing algorithms that were classified either as TP (green circles), FN (red x) or FP (red circles). On the right hand side of the figure we added summary statistics of the noisy ECG periods: the average morphSQ value, and the positive predictive values (PPV) and false negative rates (FNR) of the three detectors.

We hope this added figure supports an understanding of morphSQ and its locality and may help the reader to get a better "feeling".

ii) I strongly miss a "Related work" chapter in this work giving an overview of the state-of-the-art in ECG signal quality estimation. In the Introduction only two references are given which are from 2012 and 2007, respectively. I am absolutely not an expert in that field but I know some recent works that definitively have to be considered (e.g. https://doi.org/10.1109/tbme.2020.2969719 , https://doi.org/10.1109/rbme.2018.2810957) just by reading the relevant journals in that field.

Thank you for suggesting those two articles and the integration of an “related work” chapter. While the later issue will be focused in on of the next sections, we’d like to position ourselves to the articles you suggested. Both articles cover the topic of ECG signal quality using either the NST database or an either meta-analytic approach. Our main aim was and is the comparison of the five simultaneously worn ECG devices. The development of morphSQ was rather a byproduct to directly compare the signal quality of all devices locally and to have an indicator for the estimated ease-of-use and reliability of the ECG device. Low morphSQIs (or high values for morphSQ <10%) would indicate a rather clean signal and thus the chance-to-fail for the manufactures in-house beat-detectors would decrease. Many rather applied scientists rely on the devices automatically generated output. Insufficient data quality might lead to either lower cases numbers by excluding data from participants (or experimental parts) with low ECG quality or biased parameters computed from erroneous beat locations.
While approaches including the basic ideas of morphSQ were already existing, morphSQ can be seen as an improved version with increased locality, additional weighting, waiving the use of preceding filtering algorithms, and increased robustness due to the usage of the median instead of the mean.
While we integrated some of your later points, we added a new section to highlight those benefits of morphSQ, added related literature, and further described the aim of the development of morphSQ. You’ll find the new text fragments here (and on page 3 line 71 as well as page 8 line 238):

Part 1:

While many of the indices above require sufficient data for computation, specific methods deliver the local quality of short ECG segments: Such methods analyze the correlation between successive beat cycles (also known as template matching) [20], use spectral information [21], or calculate continuous signal-to-noise (SNR) ratios in a local time window [22]. A variety of methods are summarized in a review article by Satija et al. [23].

Part 2:

To quantify these disturbances around each single R~peak, a new local similarity-based morphological signal quality value (morphSQ) is defined. The method belongs the template-based approaches, but in contrast to the available methods it is based on the median cardiac cycle, neglects QRS distortions, and uses a weighting function to increase locality.

iii) The document structure is chaotic at various points, especially in the first chapter:
ll. 1-14: Introduction and first mention of HR and HRV
ll. 15-23: "The aim of this study is to compare usability and data quality ..."
ll. 24-41: Fundamentals of ECG (clinical vs. Holter systems, PQRST etc)
ll. 42-50: The role of HR and HRV w.r.t. different user groups
ll. 51-60: Information on the devices used
ll. 60-68: "In this study, the ECG device comparison is based on..."
ll. 69-73: Information why a high signal quality is important in research
ll. 74-80: Short summary of ECG signal quality indices
ll. 81-85: "In this paper, a new signal quality index was developed"

As I try to underline, it is very hard for the reader to follow. I highly suggest to split the introduction into i) ECG fundamentals, ii) related work, iii) the aims of this work (or research

questions), iv) and which methods will be used to reach these aims (answer the research questions).

Thank you for suggesting this new structure. We added the suggested subheadings and rearranged the text according to your proposition. Thus, the introductory part completely changed. All relevant changes can be seen in the added document: “Revised Manuscript with Track Changes.pdf”.

At some other instances of the document, the authors do not follow IMRAD. For example:
- ll. 137-143: This should be raised in the introduction or discussed at the end of the work as a limitation.

Thank you for your suggestion. We have described the devices used in the experiment in the subsection Apparatus and moved the mentioned paragraph on not using a 12-lead ECG as the gold standard to the discussion section. You’ll find the new text fragments here (and on page 14 line 459):

A limitation of this study is the missing usage of a 12-lead ECG system as a gold standard for comparison. Such a system was not applicable due to possible interferences of the chest leads with the textile electrodes of Hexoskin. Clinically validated measuring devices were used in compensation. Additionally, the focus of this comparison was not on the application for the detection of clinical abnormalities, but on the application of the devices in ergonomics and sports science, as well as in the leisure context with the focus on the valid and automated detection of R peaks in order to reduce the effort involved in manual follow-up processing steps.

- ll. 256-284: This chapter is hard to read as it mixes description of the algorithm and evaluation. I suggest to add a new subchapter "Evaluation methodology" (or similar).

Thank you for the idea to add a new subchapter. We followed your advice and added new subsections to “Statistical Analysis” in “Methods and Materials” covering the newly implemented algorithms as well as our general procedure. Due to the overlap with later comments we also added more information to the Smith-Waterman-Algorithm.

- Conclusion: All the information on usability should be part of the main body of the text (method & results). The information how long a device needs to be equipped or how the authors assess the experience needed for sensor placement is not a "Conclusion" but a result.

Thank you for highlighting this aspect. In accordance with comments of reviewer 2 we removed the more subjective information about usability of the devices completely from the paper. Thus, the manuscript should now be more stringent and focused on the objective signal quality parameters.

iv) At multiple instances the wording is not uniform. Some examples are (this list is not complete):
- l. 22: "RR intervals" vs. l. 55: "RR-intervals"
- l. 54: "PPG" (not introduced)
- l. 10: "Heart Rate (HR)" vs. l. 49: "heart rate" (abbreviation not used)
- l. 129: "EDF or EDF+" (written in italics; also not introduced) vs. Table 2: "EDF" (no italics)
- l. 210: "PQT analysis" (what is that?) vs. l. 42: "PQRST"
- l. 259: "FP - false positive" vs. l. 312: "false positive (FP)"

Thank you for mentioning this. We increased uniformity for abbreviations as far as possible. We removed the PQT analysis part. Only PQRST remained. Further doubled insertions of abbreviations were removed, missing abbreviations were inserted.
EDF and EDF+ are different iterations of the European Data Format, we added an explanation of those abbreviations to the Table 2. (For the full list of changes please see the attached document: “Revised Manuscript with Track Changes.pdf”).

Furthermore, the title does not contain the key information that in this work i) multiple, wearable ECG devices are compared and that ii) a novel method for signal quality estimation is proposed.

According to your suggestion we changed the title of our manuscript to a more specific one:
“ECG performance in simultaneous recordings of five wearable devices using a new morphological Noise-to-Signal index and Smith-Waterman-based RR interval comparisons”.
Thank you for this contribution.

v) The description of the "Smith-Waterman-like algorithm" is too superficial. I cannot understand it from the 6 lines of text and the figure provided.

We had already feared that this would become an issue. We added the description of the Smith-Waterman-Algorithm to the “used methods” section of the introduction and added a few more explanatory sentences. Please find the full description here (page 9 line 311):

Polar RR interval sequences were generated by the manufacturer's in-house algorithm, with no ability to export the underlying ECG. In this case, the pairwise comparisons of beat_ref with the exported data (query) is the direct way to assess the signal and export quality. For a fair comparison to the other ECG devices, eplimited was applied to the unsynchronized raw data (without correcting the sampling frequencies) to compute RR intervals. Since the interval data were not synchronized, a matching process had to be performed in order classify wrong or inaccurate RR intervals. In bioinformatics, the Smith-Waterman algorithm (SW)(Smith, Waterman 1981) is a well-known algorithm to perform local sequence alignment of nucleic acid sequences or protein sequences. We therefore decided to use a variation of SW to align sequences of numeric data types (RR intervals). A scoring matrix will be build that is based on gaps and individual ratings of matched pairs. The latter depends on the difference of matched RR intervals. Using dynamic programming and a traceback procedure, an optimal alignment can be derived.
Thus, an open source implementation for global alignments (https://github.com/hiraethus/Needleman-Wunsch) was modified by redefinition of a match/mismatch and its respective reward or penalty values. A modifiable reward function was defined according to our needs to perform an alignment of RR interval sequences. Equations (2) and (3) shows the definition of the scoring matrix H based on two interval sequences a and b having m and n values respectively. Function s of Equation (4) defines the reward of matched RR interval a_i from the reference sequence to the RR intervals b_j from the query sequence. Here we use a quadratic function based on the difference of both intervals in milliseconds. This defines the scoring for matched RR intervals allowing for minor deviations (reward=1 if difference is 0ms, reward=0.9 if absolute difference is 10ms, reward=0.6 if absolute difference is 20ms). The minimum value is capped by 0 and the maximum value is 1. Defining gap penalties as denoted in following Equations and in Fig. 4, it was possible to find an optimal alignment of non-synchronized interval data. Interestingly, we have rewarded a gap in the reference sequence (an insertion) by 0.9 instead of penalizing it, since in our case this is always accompanied by a

mismatch, which is already scored with 0. In this way, it was beneficial for the matching process and a better alignment could be obtained.

$$\begin{aligned} H(i,0) &= 0, \qquad & i &= 0,\ldots,m \\ H(0,j) &= 0, \qquad & j &= 0,\ldots,n \end{aligned} \tag{2}$$

$$H(i,j) = \max \begin{Bmatrix} 0 & \text{No similarity} \\ H(i{-}1,j{-}1) + s(a_i,b_j) & \text{Match/Mismatch} \\ H(i{-}1,j) - 1.0 & \text{Deletion} \\ H(i,j{-}1) + 0.9 & \text{Insertion} \end{Bmatrix}, 1 \le i \le m, 1 \le j \le n \tag{3}$$

$$s(a_i,b_j) = \max \begin{Bmatrix} 0 & \text{capped score} \\ 1 - \frac{(a_i-b_j)^2}{1000} & \text{RR interval deviation} \end{Bmatrix}, \ a_i \text{ and } b_j \text{ in ms} \tag{4}$$

The process was performed with all extracted intervals from all used devices and all experimental phases separately. An optimal alignment to maximize the overall score was found by the common traceback procedure. Using the resulting alignments of the ground truth RR intervals with the query sequences, we were to count and classify errors with a tolerance of 50 ms: gaps can be classified as either insertions (RR intervals broken up into smaller subintervals, usually treated as FP) or deletions (due to a missing beat location, usually treated as FN). Moreover,inaccurate RR intervals (due to misplaced beat locations) can be classified. Fig 4 illustrates possible findings by the matching process.

The resulting RR sequence alignment was then used to identify insertions (RR intervals splitted into smaller sub-intervals due to a false positive beat location, treated as FP) and deletions (due to a missing beat location, treated as FN) that are visible as gaps in the resulting alignment. Moreover, inaccurate RR intervals (due to misplaced beat locations) in the interval data were identified by counting the number of matchings that exceeds the tolerance of 50ms. Fig. 4 illustrates possible findings by the matching process through dynamic programming. We used the same methodology on RR interval data extracted from the raw unsynchronized ECGs of the other devices using eplimited as a standard detector for a better comparison of the quality of RR intervals (resulting from poor/excellent signal quality), see last section of Tab 5.

We hope that this changes helped to clarify how the Smith-Waterman-Algorithm works and why and how we used it to determine a signal quality index for the Polar Watch.

Minor issues:
- Please provide information how many annotators at what level of expertise provided beat_ref. Moreover, you report that arrhythmia periods were removed. Please report how this decision was made and how much data was removed.

We would like to address this point separately for the annotation and exclusion part.

In order to ensure a high data quality for the manually R-Peak correction we followed a four-eye-principle. Annotation correction was performed by two of the authors (MV, DB), both with previous experience in this field. After MV conducted a first correction, DB checked the

manually corrected files. To make this process more comprehensible for the reader we changed the statement in our manuscript (page 7 line 204):

All aligned ECG-signals were manually screened and corrected for missing, misplaced, and wrongly identified R peaks arising from the automated heartbeat detection. Following a four-eye-principle, all corrected records were screened a second time. Corrections were performed by experienced research staff.

Arrhythmia has not been identified in the 13 subjects. Only one ectopic beat has been identified in participant 10 which we would classify as blocked premature atrial contraction (See image FigA4.pdf). We also have observed respiratory sinus arrhythmia. We therefore did not removed data for generating the summary tables. We added this sentence in the “Data preprocessing -- Alignment and frequency correction” section (page 7 line 211):

Based on the procedure postulated in Vollmer 2017, we identified suspicious segments which were then manually screened. One ectopic beat was identified in the 13 participants leading to our decision to not exclude participants or ECG segments.

- Table 1: That is a strange Euro symbol? Additionally, I think it would be good to report on the bit depth of the ECG signals.

Thank you for mentioning this two points. We changed the used € sign using another LaTeX package it should look more regular now.
Depending the bit depth of the signals: This is a really valuable comment, we have not thought about before. We added it as an extra row in Table 1 named ADC (analog-to-digital-converter) precision. Please find an excerpt of the table below.

	**eMotion Faros 360°**	**Hexoskin Hx1**	**NeXus-10 MKII**	**Polar RS800 Multi**	**SOMNOtouch NIBP**
**ADC precision**	24 bit	12 bit	24 bit	NA	12 bit

- Table 2: Please define all abbreviations. What is EDF/SDC/ASC...?

We added all used abbreviations below the table and also checked again for the most commonly used ones to save the raw ECG. SDC/ASC for Faros was removed as it is not supported anymore. You can find the changes as a Note on Table 2 on page 6. Here is the list of all used abbreviations in table 2:

EDF/EDF+ -- European Data Format;
WAV -- Waveform Audio File Format;
CSV -- Comma-Separated Values;
hrm -- Polar summary export (structured text file);
RIFF -- Resource Interchange File Format;
ASCII -- American Standard Code for Information Interchange;
SCP -- Standard Communication Protocol for Computer assisted electrocardiography (SCP-ECG EN1064:2007)

- ll. 154-177: How long are P3 and P4?

Thank you for making this point. All experimental phases (P1/P2/P3/P4) lasted 5 minutes (exclusive preparation and follow-up time). We added the following statement and changed the text accordingly (page 6 lines 173):

The experimental setup was divided into four consecutive parts of five minutes each. Task order was identical for each subject to be able to determine time-on-task effects. At first, a baseline resting period was recorded while standing upright on the treadmill (P1). Upon completing P1, participants walked on the treadmill at a moderate speed (1.2 m/s) without treadmill inclination to induce medium, everyday physical workload (P2).

- l. 202: I suggest to not use the wording "Statistical analysis" as no statistical testing is performed but only mean and SD values are reported.

We have not performed inferential statistics but make use of descriptive statistics. The assessment of performance measures is part of any statistical analysis. We therefore decided not to change the section title.

- Abstract: "The results allow conclusions to be drawn..." -> I suggest to actually name the conclusions.

Due to content changes in the manuscript (removing the focus on usability) we changed the whole abstract. In this context we changed the conclusion part to a more active statement. Please find the new abstract attached:

Background: *Numerous wearables are used in a research context to record cardiac activity although their validity and usability has not been fully investigated. The objectives of this study is the cross-model comparison of data quality at different realistic use cases (cognitive and physical tasks). The recording quality is expressed by the ability to accurately detect the QRS complex, the amount of noise in the data, and the quality of RR intervals.*
Methods: *Five ECG devices (eMotion Faros 360°, Hexoskin Hx1, NeXus-10 MKII, Polar RS800 Multi and SOMNOtouch NIBP) were attached and simultaneously tested in 13 participants. Used test conditions included: measurements during rest, treadmill walking/running, and a cognitive 2-back task. Signal quality was assessed by a new local morphological quality parameter morphSQ which is defined as a weighted peak noise-to-signal ratio on percentage scale. The QRS detection performance was evaluated with eplimited on synchronized data by comparison to ground truth annotations. A modification of the Smith-Waterman algorithm has been used to assess the RR interval quality and to classify incorrect beat annotations. Evaluation metrics includes the positive predictive value, false negative rates, and F1 scores for beat detection performance.*
Results: *All used devices achieved sufficient signal quality in non-movement conditions. Over all experimental phases, insufficient quality expressed by morphSQ values below 10% was only found in 1.22% of the recorded beats using eMotion Faros 360° whereas the rate was 8.67% with Hexoskin Hx1. Nevertheless, QRS detection performed well across all used devices with positive predictive values between 0.985 and 1.000. False negative rates are ranging between 0.003 and 0.017. eMotion Faros 360° achieved the most stable results among the tested devices with only 5 false positive and 19 misplaced beat across all recordings identified by the Smith-Waterman approach.*
Conclusion: *Data quality was assessed by two new approaches: analyzing the noise-to-signal ratio using morphSQ, and RR interval quality using Smith-Waterman. Both methods deliver*

comparable results. However the Smith-Waterman approach allows the direct comparison of RR intervals without the need for signal synchronization whereas morphSQ can be computed locally.

Response to Reviewer 2:

The authors compare five different ECG devices to evaluate these devices regarding QRS detection and usability. The research question is relevant. It is also important that the authors make the recorded data publicly available. However, it exists already many papers which compare different ECG devices. Before the publication, the method should be described sufficiently detailed, as some of the reasons for the selected approach are lacking. In addition, the limitations of the method should be discussed more intensively in the discussion.

Dear Reviewer 2, thank you for taking your time to write this review. We improved our manuscript at various points and are really proud to share those improvements with you. We tried to cover all your points. Please find a detailed list of changes according to your suggestions below.
Before we start with your major and minor issues we would like to address two points you mentioned in your introductory sentences: first we would like to reinforce the uniqueness of our experimental approach, using five simultaneously worn ECG devices and second we would like to address improvements in the description of our used methods and the addition of several supplementary figures.

We added new supporting information and conducted additional analysis using a synthetic signal with artificially added noise (see Figures S3 and S4 for further information). Furthermore, a comparison of different state-of-the-art beat-detection-algorithms was added. Both approaches were combined in those supporting information/figures that are implemented to justify the relation of morphSQ to the Signal-To-Noise ratio and to beat detection performances.

Major Issues
• Introduction

Thank you for mentioning the four points below regarding our introduction. In combination with the comments of reviewer 1 we restructured the whole introduction, changed several phrases, added literature references, and tried to increase the overall stringency of our argumentation as well as clarifying the aim of our manuscript. Please find the new introduction in our revised manuscript. Thus, we will only briefly address your comments on the introduction in this letter.

o 1-4: The first reference should support this statement "[…] ongoing trend for self-quantification of personal health and fitness lead to […]" and is from 2012.

Thank you for mentioning this aspect. We added new literature to further support the statement. Please find the list of additional resources below:

10.1371/journal.pmed.1001953
https://doi.org/10.2196/34384
http://www.ncbi.nlm.nih.gov/pmc/articles/pmc5841672/

o 7-8: "A rising number of those devices are […]" here you need to add more than one reference.

We added one of the previously cited papers here again. With over 10,000,000 participants in the last years over various scientific areas wearable devices increase in importance. Especially in Figure 4 of Huhn et al. we see a large increase of studies published with commercially available wearables (<500€) in many medical fields over the last years.

https://doi.org/10.2196/34384

o 23-41: It is more important to address wearable devices instead of the Holter ECG systems.

Thank you for the good advice. We added two new papers to this part and tried to shift the focus towards newer approaches. But from our point of view, the usage of a Holter ECG system should still be preferred over some wearable devices. Especially smart watches, health rings, or in-ear-measurement-devices mainly focus on the measurement of PPG rather than writing a real ECG (which is physiologically unlikely to change). Thus, the assessment of the ECG quality is not possible. Modern approaches are currently often limited in either their sampling frequency (below 1000Hz) or decreased ability for continuous measurement and free movement (only resting measurement or momentary assessment). Future developments, especially in the area of wireless ECG technology, seem to be promising.
https://doi.org/10.1016/j.measurement.2021.109243
https://doi.org/10.1016/j.hrthm.2022.02.030

Please find the changes text part below (page 2 line 24):

At the moment most commercially available wearable devices rely on the usage of photoplethysmography (PPG) rather than writing a real ECG. Modern mobile ECG measurement solutions are often limited due to their reduced sampling frequency (below 1000Hz) or their decreased ability for continuous measurement and free movement (restricted to resting measurement or momentary assessment). Future developments, especially in the area of wireless ECG technology, seem to be promising.

o 51-60: The selection of the different ECG devices belongs to the chapter “Material and Methods” and in the chapter “Introduction”.

This is a very valid point. We removed it from the introduction section and added it in a slightly improved version to the Material and Methods section.

• Material and Methods
o The authors need to address the time synchronization of the different devices. They need to explain how they can ensure that the recorded signals are the signals for the same time interval.

The signals were previously synchronized by a multi-step approach. First, we made a ground truth annotation of heart beats based on all ECG recordings. Second, we use the annotations of R-peaks for a linear adjustment of the sampling frequencies. The signals were also aligned by using the beat intervals to correct for different starting times. Third, non-linear resampling was used to save the ECGs at a constant sampling frequency that also corrects for inaccurate and time-alternating sampling frequencies of some devices. The problems and work flow for the generation of synchronized signals are already described in a prior publication (see https://doi.org/10.22489/CinC.2019.031). We changed the introductional part of the Material and methods part and hope that this helps to solve this issue (page 7 line 196):

Necessary preprocessing steps for data alignment were performed as described in previous work (Vollmer et al. 2019). This includes the correction for time shifts as a result of asynchronous record starts and requires the correction of inaccurate and unsteady sampling frequencies. Alignment was based on the synchronization of heartbeats from a resting period by finding similar patterns in all devices. Sampling frequency was corrected by non-linear resampling to a target sampling frequency of 256 Hz (Vollmer et al. 2019).

To improve the automated signal alignment and to further evaluate the detection accuracy of each device, we generated a manual reference annotation of R peaks ($beat_{ref}$). All aligned ECG-signals were manually screened and corrected for missing, misplaced, and wrongly identified R peaks arising from the automated heartbeat detection. Following a four-eye-principle, all corrected records were screened a second time.

o Subjects: Please add the inclusion and exclusion criteria for the subjects.

Thank you for mentioning this point. We changed the subject description accordingly. We were able to include all participants for whose the biometric Hexoskin Shirt would fit (only limited sizes were available) and had to exclude participants with known cardiovascular diseases. Please find the text changes here (page 6 line 161):

13 healthy participants (six male) in the age range between 21 and 35 years (μ = 28.00, σ = 4.28) took part in the experiment. Due to limited sizes of the biometric Hexoskin shirt only participants could be included for whose a fitting size was available. Further participants had to confirm that no prior cardiac problems or diseases are known.

o Statistical analysis: Please add the explanation why you chose the algorithm eplimited and did not choose another state-of-the-art algorithm (e.g., template matching or deep learning approach).

To address this topic we added two figures to this letter and performed some additional analysis (Fig. 5001 and A2). In a first step 13 different beat detection algorithms were compared for their overall FNR during all four experimental phases of our dataset. In addition to widely used algorithms (wqrs, eplimited, pantomkins, hamilton, engelsezeelenberg) newer approaches like the envelope-based rdeco (https://doi.org/10.13026/x6j7-sp58, https://doi.org/10.7717/peerj-cs.226), or neural-network-based (LSTM) ecg2rr (https://doi.org/10.1145/3341105.3373945) were used. Figure A1 and A2 illustrate the results of these comparisons. Best performing algorithms were eplimited, engelsezeelenberg, and pantomkins_tma.

Description Figure A1:
Figure A1 shows the average performance of heart beat detection (average across 13 participants and 4 signals, 50ms tolerance, expressed by FNR and PPV) of 13 different algorithms in each experimental phase that were applied to these signals: 'NEXUS_Sensor_B_EKG', 'HEXOSKIN_ECG_I', 'FAROS_ECG', 'SOT_aVF' (ECG-signal-names as used in: https://doi.org/10.13026/chd5-t946), which turned out to be the signals with the best performance rates. The scatter plot shows the rates on log-scale where eplimited, engelsezeelenberg, and pantomkins_tma showed best performance even in the running phase.

Description Figure A2:

Figure A2 presents a detailed view of Fig. A1, focusing on the area of FNR and 1-PPV up to approximately 1%. Only eplimited, engelsezeelenberg, and pantomkins_tma could be mapped for all four experimental phases and the combined overall illustration. While for the whole duration of the experiment (covering all phases + waiting times) all three algorithms performed similar (FNR and 1-PPV close to 1%) differences during the resting phase can be seen, which should be the phase with the lowest noise percentage (at least close to zero movement artifacts). Overall eplimited still seems to perform best between those three algorithms. Details for the detection accuracy during noise parts can also be seen in figures S3 and S4 (see answer to Reviewer 1 for a detailed explanation of those figures). FNR and 1-PPV of around 1% seem to be comparatively low (Fig. A1 indicates that a few algorithms perform pretty poor resulting in values above 10%) but a manual revision/correction of the detected R-beats is still highly recommended. High signal quality (as expressed by morphSQ) would decrease the chance for human errors during this process because it is easier to identify R-Peaks.

Thus, eplimited outperformed newer algorithms like r-deco or ecg2rr such that we have i9ncluded results with eplimited as the primary algorithm of our choice as it was already presented in the manuscript. Anyway, sufficient signal quality is expressed by low errors rates independently of the chosen detector.

o 278: It is important to use the same algorithms to make the results comparable.

This is a very valid point, thank you for mentioning it. We had to include the Polar watch in our comparison to cover more wearable devices which often lack in the ability to export a raw ECG. Most wearable devices support the option to export RR intervals, which should be the minimum requirement for the use in scientific research from our point of view. Thus, the our SQI approach was not applicable on the Polar watch. Using the Smith-Waterman-Algorithm we included an additional approach to quantify the data quality of the detected RR intervals. To increase consistency and make the results more comparable the Smith-Waterman-Algorithm was used to analyze the RR interval quality for the remaining four devices too. Therefore we used the raw ECG signals and the RR intervals derived from the usage of eplimited. Results were added to the new Table 5. Please find the table below:

Table 5. QRS detection performance through RR interval alignment using the modified Smith-Waterman algorithm for numericals. Errors are expressed by false positive counts (FP), false negative counts (FN), and the number of misplaced annotations from unsynchronized data (50 ms tolerance).

	Overall	P1 – Rest	P2 – Walking	P3 – 2-back	P4 – Uphill
**FP|FN|Misplaced using Smith-Waterman approach***					
eMotion Faros 360°	5|0|19	0|0|0	0|0|0	0|0|0	0|0|6
Hexoskin Hx1	85|10|765	3|0|5	114|0|204	11|0|14	29|1|270
NeXus-10 MKII	40|5|765	0|0|0	3|0|4	15|0|40	0|1|309
SOMNOtouch NIBP	63|0|202	0|0|0	8|5|134	0|0|2	0|0|65
Polar RS800 Multi	28|29|333	0|3|18	6|0|57	3|3|66	0|0|11

* Comparison based on exported Polar RR intervals and detected heart beats (eplimited) in raw unsynchronized ECGs.

We also added a new paragraph covering Smith-Waterman and its usage to the methods section. We hope that those changes increase the internal consistency of our analysis and helped to remove your concerns.

• Conclusion

o 401 „Fig 6. Authors' ordinal ranking of used recording devices.“: This ranking depends on the author's opinion and is not scientific. The authors need to exclude this figure.

Thank you for mentioning this aspect. When we wrote the first draft we were strongly focusing on usability aspects. Thus, we included this ranking figure. With the improvements and the focus shift towards a more methodological aspect, we totally agree with you and excluded the figure and the corresponding text.

Minor Issues
• Figures and tables
o Table 1: You should replace the euro signs with numbers.

Thank you for mentioning this point. We tried to implement this point, but unfortunately during this process several layout changes in the table occurred due to several new lines. Thus, we decided to contain the euro signs in the table. But according to a note of reviewer 1 we changed the used €-sign in the table, so we hope that this increased the readability and this issue is no longer too disturbing.

o Figure 5: It is important to differentiate between the different use cases and to explain the results.

Thank you for this suggestion. We changed the corresponding text and added further information. Please find the complemented paragraph below (and on page 12 line 424):

Further, F1 scores are used to describe accuracy of detected heartbeats on the participant level. Thus, the influence of single participants can be shown (Fig 5). An F1 score below 90% is considered as an insufficient ECG quality that results in increased error probability during automated beat annotation processes. This is the case for one participant measured with NeXus during P4 and in one participant measured with Hexoskin during walking (P2) but but not while running (P4). Overall, Polar had the most scores low or insufficient quality (below 99%: 7 participants), mainly attributable to the 2-back task, but achieves very high scores during the running phase. Using SOMNOtouch and Hexoskin one respectively three participants had low F1 scores.

Please find the additional paragraph in the discussion (page 14 line 446):

Five devices made for specific target groups were tested. Holter ECG systems, chest straps, and biometric shirts all reached acceptable results with use case specific accuracy. This is supported by the low number of insufficient quality periods (see Fig. 5). The medical device Faros achieved almost perfect results even during movement phases. Hexoskin's main problem is the dependency of the shirt size to fit the participant perfectly. Sensor placement may change during body movement and may result in reduced accuracy. NeXus overall accuracy is sufficient with deficits in the uphill condition. There is one participant for whom NeXus produces unusable data (c.f. Fig. 5). If this participant is excluded, NeXus reaches signal quality (morphSQI(P3)=0.015, morphSQI(P4)=0.028) comparable to Faros and SOMNOtouch. Polar, as the most popular mass-producing sports equipment manufacturer of the devices used, achieved the best results in phases with higher movement and thus higher HR. Nevertheless statistical differences has not been conducted due to the low number of participants.

Response to Reviewer 3:

Dear Reviewer 3, thank you very much for taking the time to comment on our manuscript. We thoughtfully tried to integrate your points in our new revised version of our manuscript. Please find our comments and adjustments below.

- The authors introduce a novel signal quality index. I think it would make a lot of sense to either evaluate this signal quality index on existing benchmark problems first or to evaluate their data using also other signal quality indices and see whether or not there are any differences, if their new metric correlates with other metrics, etc.

Thank you for your valuable comment. Indeed we missed some comparisons that motivates and justifies our new approach. We conducted simulations to show relations to the detectability of R peaks using standard algorithms and to show the relation to known Signal-To-Noise ratios from semi-artificial ECGs, described as follows:

We added new supporting information and conducted additional analysis using a synthetic signal with artificially added noise (see Figures S3 and S4 for further information). Furthermore, a comparison of different state-of-the-art beat-detection-algorithms was added to address reviewer 2's suggestions. Both approaches were combined in those supporting information/figures.

Figure S3 and S4 summarize the results from the application of the morphSQ-methodology on synthetic signals of the PhysioNet Noise Stress Test (NST) database (https://doi.org/10.13026/C2HS3T). This database contains two original records (total length 30 minutes each) with realistic noise added to clean signals at specific time periods (alternating every two minutes). The ground truth beat annotation files from the clean recordings was used to evaluate the PPV and FNR. We have decreased the step-length to 1 db of the pre-defined signal-to-noise ratio bandwidth (originally 24 to -6 db with a 6 db step-length) to increase resolution, and improve interpretability through the generation of additional noisy records by applying the nst-function (from the WFDB toolbox, https://doi.org/10.13026/egpf-2788) to 100 relatively clean records of the set-p training database of the PhysioNet/CinC Challenge 2014 (see methodology in https://doi.org/10.22489/CinC.2017.182-178, Challenge data and description: https://doi.org/10.1088/0967-3334/36/8/1629, https://lcp.mit.edu/pdf/MoodyCinC2014.pdf , https://physionet.org/content/challenge-2014/1.0.0/).

Based on the suggestion of Reviewer 2 additional beat-annotation-algorithms were included in the analysis. In a first step 13 different beat-annotation-algorithms were compared for their overall FNR during all four experimental phases of our dataset. In addition to widely used algorithms (wqrs, eplimited, pantomkins, hamilton, engelsezeelenberg) newer approaches like the envelope-based rdeco (https://doi.org/10.13026/x6j7-sp58, https://doi.org/10.7717/peerj-cs.226), or neural-network-based (LSTM) ecg2rr (https://doi.org/10.1145/3341105.3373945) were used. Figure A1 and A2 illustrate the results of these comparisons and are only added for this letter. Best performing algorithms were eplimited, engelsezeelenberg, and pantomkins_tma, those are included in the noise-related analysis (as depicted in Fig. S3 and S4).

Description of Fig S3:
Figure S3 is composed of two parts. Part S3a shows the beat detection performance of the three best-performing algorithms expressed by false discovery rates (FDR) and false negative rates (FNR). Part S3b illustrates the corresponding performance of morphSQI based on reference annotations. Analysis were based on 100 recordings (extended from set-p database) with

increasing amount of noise for clean parts and noisy parts separately (left and right column of the figure). The average morphSQI was computed from the same recordings and the relation to the SNR can be observed in the accuracy measures of the three algorithms and in morphSQI at the same time. Correlations of morphSQI with FDR and FNR were further investigated results can be seen in Fig S4.

Description of Fig S4:
Figure S4 show the correlation of the new signal quality metric morphSQ with the beat detection performance of the three detection algorithms evaluated from the noise stress test recordings. We have noticed that for some of the recordings the algorithm from Engelse-Zeelenberg and the modified Pan-Tompkins were not able to detect any beat. This resulted in an empty annotation file and hence FDR and FNR cannot be computed. As all beats are missed, we set FDR and FNR to 100% and marked them as gray dots in the scatter plots. Spearman's rank correlation coefficient r_S was computed between morphSQI and FDR/FNR values of eplimited. With a FDR-correlation of r_S = .969 and a correlation to FNR of r_S = .949 a strong relation of beat detectability and morphSQI can be assumed. Compared to FDR and FNR morphSQ has the advantage to be possibly computed locally (from a few beats only) whereas the estimation of FDR and FNR usually rely on at least dozens of beats. Following these observations and analysis we assumed an increased reliability and robustness of eplimited and thus used it for further analysis of our experimental data (see Tab. 4).

Further a short paragraph was added to the manuscript covering this topic (see page 9 line 287):

For a better understanding of morphSQ, Fig 2 shows different ECG samples and the corresponding morphSQ (additional cases are illustrated in Fig S2). Using clean ECG recordings and realistic noise measurements from PhysioNet we investigated the performance of beat detection and morphSQ in relation to signal noise. Fig S3 shows an example ECG from the Noise Stress Test database, where realistic noise was added to a clean record. morphSQ has been computed at each beat and the performance of beat detection in clean and noisy ECG sections was evaluated. The relation of average morphSQ with increasing SNR ratios was investigated using the methodology as described in Vollmer 2017. Fig S4 summarizes the results and shows the direct relation to NSR. The similarity-based signal quality expressed by morphSQ is sensitive to beat misplacements and therefore requires accurate, complete, and verified beat annotations.

(as a side note, I find it somewhat unintuitive that the signal quality index has to assume low values for the signal to be of good quality. In Figure 3, the authors even write "quantify similarity", so why not something like "1 – SQI"? Then, the authors also don't need to talk about "Noise to Signal" in their manuscript but rather SNR...)

Thank you for this side note. We added a sentence featuring this problem, stating that we just used the standard nomenclature of the SQI research to indicate that morphSQ is made to express something similar to the standard SQIs. Additionally, we changed the figure text to "Quantify divergence". Please see the added sentence below (page 9 line 283):

Defining morphSQ as a NSR rather than a signal-to-noise ratio (SNR) increases the ease of use and interpretability. Lower values indicate a smaller amount of noise with a minimum of 0%, while at the same time the usage of the percentage scale is easier to interpret (even for inexperienced users) than the usual decibel/db scale for SNR.

We decided to stay with the SQI nomenclature and did not change the mathematical basis following this two points:

1. The unit of morphSQ is in percent (%) and represents the estimate of the (weighted) average noise amplitude. The minimum value of morphSQ is 0%. But the values can exceed 100%, when having noise in the signal that has a larger amplitude than the underlying clean physiological ECG. 1-morphSQ would therefore be also unintuitive because it can have negative values.
2. Using signal quality indexes in db (decibel) is rather complex to understand for many applied scientists. Using morphSQ on a percentage scale, we tried to implement an index that focuses on the total amount of noise in comparison to the clean signal making it easier to understand and interpret even for mathematically less experienced readers. To help the reader to understand morphSQ and the dependency to the Signal-To-Noise ratio figure S4 was added.

We are currently working on an additional short-paper using morphSQ as a basis for a signal quality index in db.

- The SQI cannot be applied to the Polar-device for obvious reasons. However, this makes the analysis somewhat inconsistent. Thus, I believe the authors should make a much stronger point on why to include this device (other than because it was there) or think about leaving it out.

Thank you very much for pointing this out. We tried to improve our manuscript at this point in two ways: first we added a short paragraph about the necessity to include devices like the polar watch in the (new) introduction part under aims of this study. An increasing amount of studies in various research fields (see https://doi.org/10.2196/34384) is conducted using mobile devices like the polar watch we used. For most of them the export of the full ECG (more correct for most watches, rings, and in-ear-devices the PPG) is not possible, but for research purposes they should at least provide RR intervals. Adding the Polar watch to our comparison enables us to additionally analyze the quality of RR interval exports (also Hexoskin provides such processed data) in comparison to manually extracted beat locations from raw ECGs. These additional analyzes do add valuable information to the community.
To increase consistency and making the results more comparable, the Smith-Waterman-Algorithm was used to analyze the RR interval quality for the remaining four devices too. Therefore we used the raw ECG signals and the RR intervals derived from the usage of eplimited. Results were added to the new Table 5. An excerpt of the table can be found below:

Table 5. QRS detection performance through RR interval alignment using the modified Smith-Waterman algorithm for numericals. Errors are expressed by false positive counts (FP), false negative counts (FN), and the number of misplaced annotations from unsynchronized data (50 ms tolerance).

	Overall	P1 – Rest	P2 – Walking	P3 – 2-back	P4 – Uphill
**FP|FN|Misplaced using Smith-Waterman approach***					
eMotion Faros 360°	5|0|19	0|0|0	0|0|0	0|0|0	0|0|6
Hexoskin Hx1	85|10|765	3|0|5	114|0|204	11|0|14	29|1|270
NeXus-10 MKII	40|5|765	0|0|0	3|0|4	15|0|40	0|1|309
SOMNOtouch NIBP	63|0|202	0|0|0	8|5|134	0|0|2	0|0|65
Polar RS800 Multi	28|29|333	0|3|18	6|0|57	3|3|66	0|0|11

* Comparison based on exported Polar RR intervals and detected heart beats (eplimited) in raw unsynchronized ECGs.

We hope that those changes increase the internal consistency of our analysis and helped to remove your concerns.

Additionally we added a paragraph on Polar usage below (page 4 line 100):

Despite the fact of a missing raw ECG export functionality, the implementation of wearables in scientific research areas is risin (Huhn et al. 2022). Adding Polar to the comparison enables us to additionally analyze the quality of RR interval exports, a widely used feature even for consumer products.

Additionally we added a paragraph on the Smith-Waterman-based comparisons (page 9 line 311):

Polar RR interval sequences were generated by the manufacturer's in-house algorithm, with no ability to export the underlying ECG. In this case, the pairwise comparisons of beat_ref with the exported data (query) is the direct way to assess the signal and export quality. For a fair comparison to the other ECG devices, eplimited was applied to the unsynchronized raw data (without correcting the sampling frequencies) to compute RR intervals. Since the interval data were not synchronized, a matching process had to be performed in order classify wrong or inaccurate RR intervals. In bioinformatics, the Smith-Waterman algorithm (SW)~\cite{smith1981identification} is a well-known algorithm to perform local sequence alignment of nucleic acid sequences or protein sequences. We therefore decided to use a variation of SW to align sequences of numeric data types (RR intervals). A scoring matrix will be build that is based on gaps and individual ratings of matched pairs. The latter depends on the difference of matched RR intervals. Using dynamic programming and a traceback procedure, an optimal alignment can be derived. Thus, an open source implementation for global alignments (https://github.com/hiraethus/Needleman-Wunsch) was modified by redefinition of a match/mismatch and its respective reward or penalty values. A modifiable reward function was defined according to our needs to perform an alignment of RR interval sequences

- Along the same lines, the authors make an argument as to why they are not using a 12-lead ECG as reference (which is plausible) while, at the same time, argue that their SQI might be useful in the context of “further PQT analysis”. This seems like an ambiguity to me.

Thank you for mentioning this topic. We removed a part of the sentence originally containing the PQT analysis and added a short paragraph to the conclusion focusing on potential application of morphSQ to p- and t-wave analysis. The aim of this sentence was just to show the potential of morphSQ besides R-Peak detection. Please find the added paragraph below (page 15 line 531):

morphSQ uses an intuitively interpretable percentage scale and works perfectly on a local level. It thus shows high correlation to SNR, FNR, and PPV of the detection of R~peaks, but requires manually revised annotations of R~peaks for reliable values. A future application of the morphSQ algorithm might be the additional analysis of p- and t-wave autocorrelation.

- I find the presentation of results in the large tables somewhat confusing. The authors could think about, for example, highlighting the best values, etc. Also, is there a way to perform some sort of statistical test to see whether or not the results of the different devices and test scenarios are significantly different?

To avoid confusions, we have split the original Table 4 into Table 4 (showing results on beat detectability from aligned signals) and Table 5 (showing results from RR interval alignment with our Smith-Waterman-approach). We would like to avoid highlighting because the reader should not be biased by our decision on what we highlight. Also, the results are very close given the

overall number of beats that would make it hard for us to set any threshold. On the other hand we provided Figure 5 that shows the performance in all 13 participants. Minor but statistically significant differences in the F1 value, e.g. showing that the F1 values of Faros and Nexus significantly differ, would not be meaningful as the quality and usability of the records are still regarded sufficient. Additionally, due to the small sample size a categorical comparison using the classes (comparing counts from excellent/green, low quality/yellow, poor/red) cannot be conducted.

- The conclusion section is rather long and also introduces a new figure, I would consider rearranging the content between discussion and conclusion.

Thank you for this suggestion. Following the additional input from reviewer 2 we decided to removed the figure and the corresponding text from the conclusion. Thus, the section is now shorter and more stringent.

- The paper could benefit from another round of proof reading from a native speaker ”differences in usability and practicability between all devices ARE characterized”, “As it was the goal to induce a mental state” (?). Also, it seems to me that punctuation could be improved.

We tried to improve the paper using a non-specialized proofreader and changed several language issues (punctuations, spelling, word order). A full list of changes can be found in the added document “Revised Manuscript with Track Changes.pdf”

- “During a first check of the recorded data from all five devices, several problems occurred that needed to be solved.” I am not sure this is a good sentence to start the paragraph as this should be a result-oriented (and not a progress-oriented) report.

Thank you making this suggestion. We totally agree with you and changed the paragraph to the following (page 7 line 196):

Necessary preprocessing steps for data alignment were performed as described in previous work (Vollmer et al. 2019). This includes the correction for time shifts as a result of asynchronous record starts and requires the correction of inaccurate and unsteady sampling frequencies. Alignment was based on the synchronization of heartbeats from a resting period by finding similar patterns in all devices. Sampling frequency was corrected by non-linear resampling to a target sampling frequency of 256 Hz (Vollmer et al. 2019).
