# Supplementary material for: ECG performance in simultaneous recordings of five wearable devices using a new morphological noise-to-signal index and Smith-Waterman-based RR interval comparisons
Source: PLoS One. 2022 Oct 5;17(10):e0274994. doi: 10.1371/journal.pone.0274994 (PMC9534432; doi:10.1371/journal.pone.0274994)
Supplement: Attachment — Submitted filename: Response to Reviewers.pdf [file pone.0274994.s007.pdf]

Response to Reviewers

Dear Reviewer 3,

thank you very much for taking your time again to review our improved manuscript. We highly appreciate your decision and tried to further improve the manuscript but adding the paper you suggested. Please find the new parts where we referred to the paper below.

(page 3, line 71-78)

While many of the indices above require sufficient data for computation, specific methods deliver the local quality of short ECG segments: Such methods analyze the correlation between successive beat cycles (also known as template matching) [20], use spectral information [21], or calculate continuous signal-to-noise ratios (SNR) that are based on local time windows and noise-free signals, e.g., computed through low-pass filtering or wavelet decomposition [22]. In contrast, Hoog Antink et al. [23] defined a shape-based SNR (SNRs) using cardiac templates for the approximation of noise-free signals

(page 8, line 240-242)

The method belongs the template-based approaches, such as SNRs [23], but in contrast to the available methods it is based on the median cardiac cycle, neglects QRS distortions, and uses a weighting function to increase locality.

Thank you again for reviewing our manuscript.
